# Two-year Prevalence of Minor Aphtha in Tabriz, Northwest Iran

**DOI:** 10.5681/joddd.2009.005

**Published:** 2009-03-16

**Authors:** Mahmood Sina, Mahmood Toorchi, Sepideh Vosough Hosseini, Ali Taghavi Zenouz, Masoumeh Mehdipour

**Affiliations:** ^1^Assistant Professor, Department of Oral and Maxillofacial Pathology, Faculty of Dentistry, Tabriz University of Medical Sciences, Tabriz, Iran; ^2^Associate Professor, Department of Biotechnology, Faculty of Agriculture, Tabriz University, Tabriz, Iran; ^3^Associate Professor, Department of Oral and Maxillofacial Pathology, Faculty of Dentistry, Tabriz University of Medical Sciences, Tabriz, Iran; ^4^Assistant Professor, Department of Oral Medicine, Faculty of Dentistry, Tabriz University of Medical Sciences, Tabriz, Iran

**Keywords:** Age, minor aphtha, sex, smoking

## Abstract

**Background and aims:**

Recurrent aphthous stomatitis is a condition comprised of oral painful ulcers appearing at inter-vals in different intraoral sites, triggered by a variety of causative agents in certain subgroups of patients. Since there are no studies on the subject in Northwest Iran, the aims of the present study were to evaluate the prevalence of aphthous ulcer and to assess the association of some influencing factors on minor aphtha.

**Materials and methods:**

Of all patients examined during a two-year period, 33 patients were diagnosed with aphthous lesions. A questionnaire was used to collect the data including age, gender, familial history, smoking habit, and food allergy of the patients. Chi-square test was used to assess the association of variables.

**Results:**

The prevalence of aphthous lesions was found to be 0.3%, and was significantly higher in females compared with males (23 females and 10 males, respectively; P = 0.024). Familial involvement of aphthous ulcer was reported in 42.4% of the patients (P = 0.411). The aphthous ulcer was seen less frequently in smokers compared with non-smokers (P = 0.024).

**Conclusion:**

A relatively low prevalence of minor aphtha was found in the studied population. Higher prevalence in females and non-smokers were observed.

## Introduction

Recurrent aphthous stomatitis is the most common oral recurrent ulcer affecting 10% to 20% of the popu-lation. ^1 - 3^ The reported prevalence of aphtha is 5% to 66% with a mean of 20%. ^4^ It is comprised of oral painful ulcers appearing at intervals in different in-traoral sites, triggered by a variety of causative agents in certain subgroups of patients. The clinician should explain the diverse underlying causes to the patient, noting that the most exhaustive search for the causes may lead to an elusive answer. Several hypotheses including autoimmunity, food allergy, hematological disorders, heredity, psychological stress, viral infec-tions, immunodeficiency, and local trauma have been proposed as initiating factors of aphthous lesions.

Many specialists and investigators in oral medicine no longer consider aphtha to be a single disease but, rather, several pathologic states with similar clinical manifestations. ^2^ Research has shown normal hemo-globin and blood cell count rate in patients with aphthous ulcers. ^5^ The cause appears to be “different things in different people” and no single triggering agent is responsible. ^4 , 6^

Since there are no studies on the subject in Northwest Iran, the aims of the present study were to evaluate the prevalence of aphthous ulcer and to assess the influence of age, gender, familial history, food allergy, and smoking on minor aphtha.

## Materials and Methods

**Figure 1:**
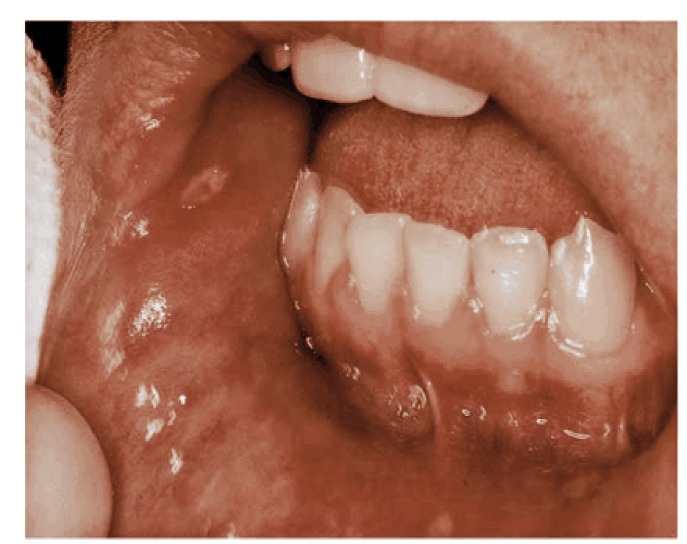
A typic example of minor aphtha.

The study population included patients presenting the Department of Oral Medicine, Tabriz University of Medical Science, Tabriz, Northwest Iran, between May 2002 and October 2004, with oral ulcer as their chief complaint. Patients were examined by an oral medicine specialist and the history of the lesion was carefully evaluated. The diagnostic criteria for minor aphtha included lesions that demonstrated a whitish membrane encircled by an erythematous halo measuring between 3 and 10 mm in diameter, which healed without scarring in 7 to 14 days.^4^ A typical instance of minor aphtha is shown in Figure 1. A questionnaire was designed to collect information including age, gender and intra-oral site of involvement as wells as subjects’ history of familial involvement and smoking. The questionnaire was filled by the same clinician who examined the patients. Patients who had used medications in the last three months were excluded from the study.

Data were analyzed using SPSS 13.0 computer software. Descriptive statistics were used to report the prevalence of aphthous lesions. To determine statistical association of prevalence of lesions with gender, familial involvement, food allergy and smoking, chi-square test was used.

## Results

Of 11100 patients examined, a total of 33 patients, 10 males (30.3%) and 23 females (69.7%), were diagnosed with minor aphtha. The prevalence of aphthous lesions was calculated as 0.3% and was higher in females than in males (P = 0.024). Aphthous lesion was seen more between 20 to 40 years of age (P < 0.1, Table 1). Familial involvement of aphthous ulcer was reported in 42.4% of the patients (P = 0.411). There was no significant association between positive history of food allergy and occurrence of minor aphtha (P = 0.232). Positive history of smoking was seen in 30.3% of the patients who had a lower occurrence of aphthous lesions compared with non-smokers (P = 0.024). Minor aphtha involved tongue, lip and buccal mucosa more than other sites in the oral cavity.

## Discussion

The prevalence of minor aphtha in this study was found to be 0.3%. This is a rather unexpected finding of the present study, as the prevalence of aphtha has been reported between 5–66% previously.^1-4^

A finding of this study that shows 69.7% of minor aphtha cases were females is in accordance with the results of several other studies that have reported higher incidence of minor aphtha in females.^1,2,4^

Previous studies have shown that minor aphtha occurs mostly in the second and third decades of life;^1,2^ however, in the present study, the occurrence of minor aphtha was seen slightly more in the third and fourth decades of life (Table 1). In addition, minor aphtha has been reported among infants in Italy.^7^ Aphthous ulcerations are noted more frequently in children and young adults,,^8^ but the annual incidence in adults younger than 40 years old is almost twice that of older adults.^4^ A similar pattern was observed in the present study.

**Table 1:** Distribution of patients based on age group

Decade of life	1^st^	2^nd^	3^rd^	4^th^	5^th^	6^th^	7^th^
Number of patients	4	2	10	6	5	5	1

Food allergy has been regarded as an initiating factor of minor aphtha.^1^ However, there was not any significant association between positive history of food allergy and occurrence of minor aphthous lesions in the present study (P = 0.232). Despite the proposed possibility of food allergy acting as a predisposing agent, the lack of association with the occurrence of this lesion in this study may be related to genetic attributes of the studied population, as food allergy may be part of the genetic endowment.^1,4^ However, although in the studied samples food allergy has not influenced the ulcers, the precise cause of this finding can be subject of further research admitting a higher sample size.

Familial history has an influence on the incidence of aphtha.^9,10^ In this study, however, there were no significant associations between the prevalence of minor aphtha and a positive familial history (P = 0.411), which is in agreement with the results of a previous study.^8^

In the present study, there was a significant association between the occurrence of aphtha and smoking habit (P = 0.024), which is in line with the results of previous studies.^2,4,11^ The lower occurrence of aphthous lesions in smokers could be explained by the fact that hyperkeratosis of the squamous stratified epithelium in smokers might serve as a barrier against minor aphtha in the oral mucosa.^4^

## Conclusion

During the study period, the prevalence of minor aphtah in the study population was found to be 0.3%. Females were more susceptible than males. Minor aphtha was more frequently seen between 20 to 40 years of age and was seen less frequently in smokers. It was not associated with a positive familial history or food allergy.
